# Supplementary material for: Characterisation of the Semliki Forest Virus-host cell interactome reveals the viral capsid protein as an inhibitor of nonsense-mediated mRNA decay
Source: PLoS Pathog. 2021 May 21;17(5):e1009603. doi: 10.1371/journal.ppat.1009603 (PMC8174725; doi:10.1371/journal.ppat.1009603)
Supplement: S1 Fig — Geldoc images of coomassie-stained gels showing eluates (20 μL) from three biological replicates of each SFV affinity purification electrophoresed 1 cm into the gels, prior to cutting. Rectangular segments (10 mm x 3 mm) for each lane were cut from the gel and samples were processed for mass spectrometry analysis. Note that samples of Rep1: nsp3-Z were loaded in opposite positions, hence highlighted in red and denoted (+) and (-). (PDF) [file ppat.1009603.s001.pdf]

Supporting information: Fig S1

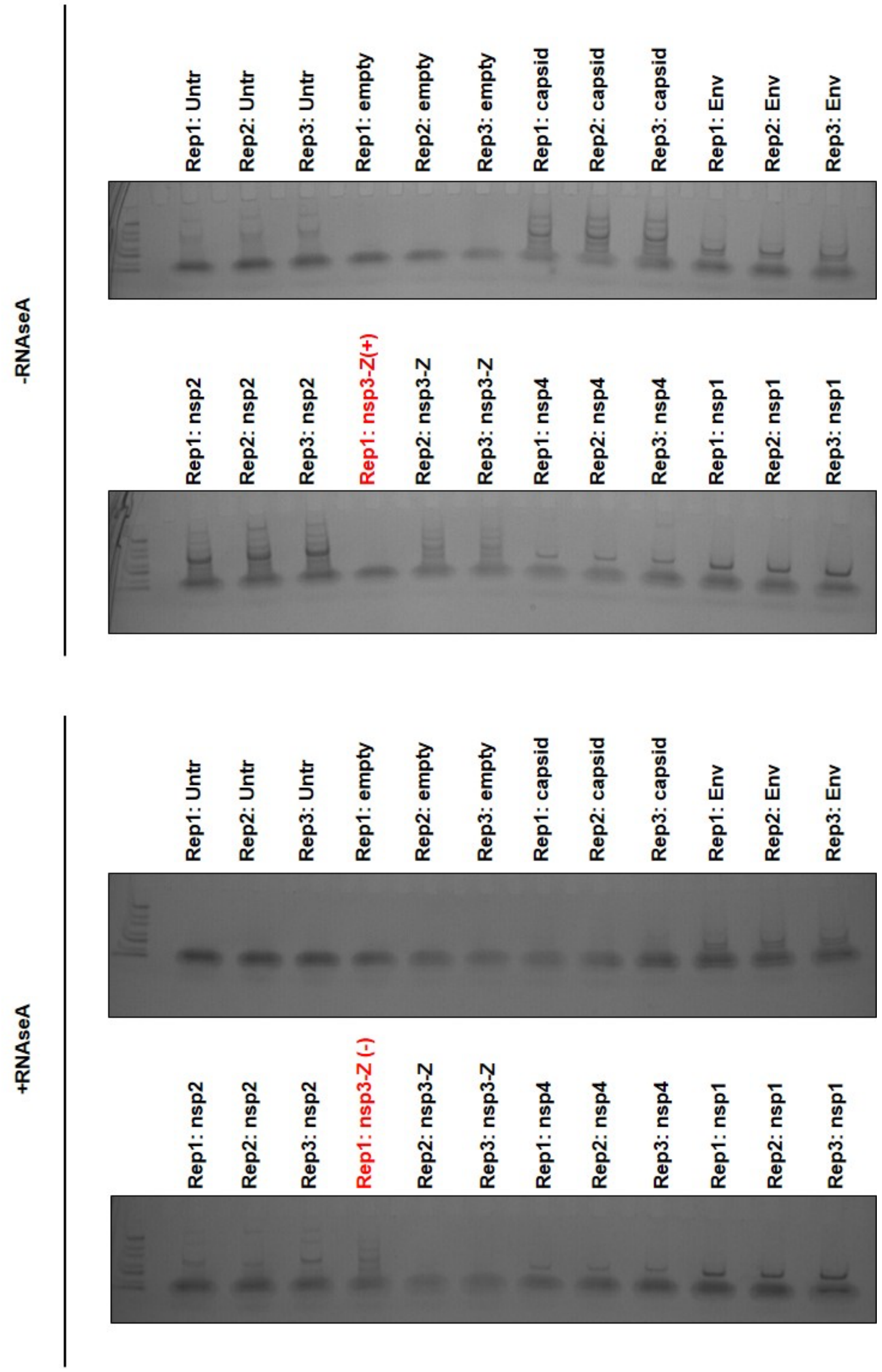

Figure S1: Coomassie-stained gels showing eluate samples of SFV affinity purifications that were sent for mass spectrometry analysis. Geldoc images of coomassie-stained gels showing eluates (20 µL) from three biological replicates of each SFV affinity purification electrophoresed 1 cm into the gels, prior to cutting. Rectangular segments (10 mm x 3 mm) for each lane were cut from the gel and samples were processed for mass spectrometry analysis. Note that samples of Rep1: nsp3-Z were loaded in opposite positions, hence highlighted in red and denoted (+) and (-).
