# Supplementary material for: Characterisation of the Semliki Forest Virus-host cell interactome reveals the viral capsid protein as an inhibitor of nonsense-mediated mRNA decay
Source: PLoS Pathog. 2021 May 21;17(5):e1009603. doi: 10.1371/journal.ppat.1009603 (PMC8174725; doi:10.1371/journal.ppat.1009603)
Supplement: S2 Fig — Anti-flag western blots of SFV affinity purifications with (bottom) and without (top) treatment with RNaseA. Red asterisks indicate 3xFLAG tagged SFV proteins (or the 3xFLAG alone) at respective sizes. ‘Untr’ refers to an untransfected control condition that underwent the affinity purification procedure, while ‘empty’ denotes transfection with a plasmid construct containing only the 3xFLAG tag (with no additional coding region) followed by affinity purification. The expected sizes of the proteins (3xFLAG included) were: empty ~8kDa; nsp1 ~63kDa; nsp2 ~92kDa; nsp3-Z ~82kDa; nsp4 ~72kDa; capsid ~33kDa and Env ~111kDa. The left side of the WBs indicate the Eluates (after flag peptide elution from the beads) of each SFV affinity purification, which were sent for mass spectrometry analysis. The right hand side of the WBs indicate the SFV proteins that were still present on the beads after the flag peptide elution step. (PDF) [file ppat.1009603.s002.pdf]

Supporting information: Fig S2

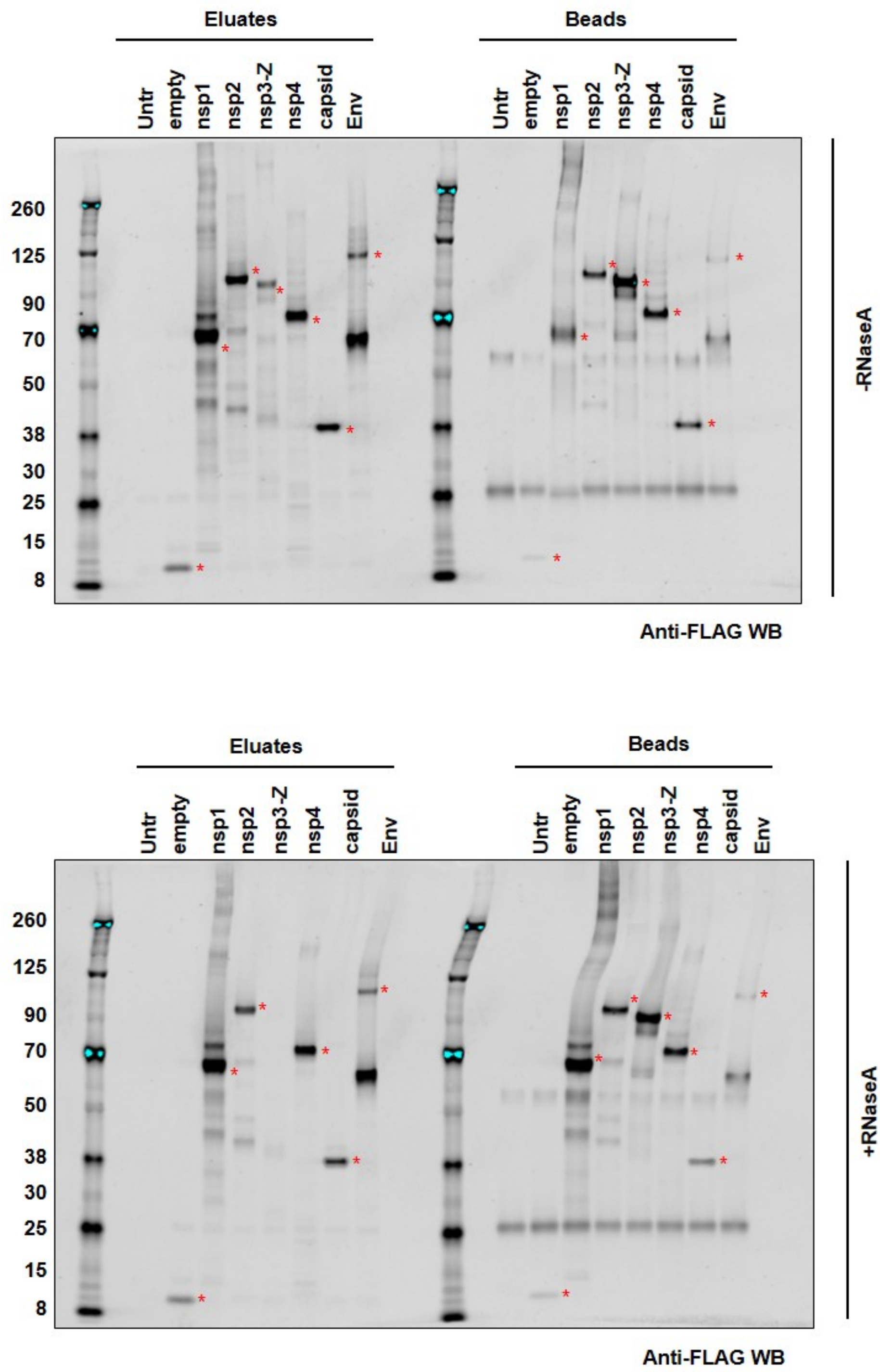

Figure S2: Anti-flag western blots of SFV affinity purifications with (bottom) and without (top) treatment with RNaseA. Red asterisks indicate 3xFLAG tagged SFV proteins (or the 3xFLAG alone) at respective sizes. 'Untr' refers to an untransfected control condition that underwent the affinity purification procedure, while 'empty' denotes transfection with a plasmid construct containing only the 3xFLAG tag (with no additional coding region) followed by affinity purification. The expected sizes of the proteins (3xFLAG included) were: empty ~8kDa; nsp1 ~63kDa; nsp2 ~92kDa; nsp3-Z ~82kDa; nsp4 ~72kDa; capsid ~33kDa and Env ~111kDa. The left side of the WBs indicate the Eluates (after flag peptide elution from the beads) of each SFV affinity purification, which were sent for mass spectrometry analysis. The right hand side of the WBs indicate the SFV proteins that were still present on the beads after the flag peptide elution step.
