# Supplementary material for: Characterisation of the Semliki Forest Virus-host cell interactome reveals the viral capsid protein as an inhibitor of nonsense-mediated mRNA decay
Source: PLoS Pathog. 2021 May 21;17(5):e1009603. doi: 10.1371/journal.ppat.1009603 (PMC8174725; doi:10.1371/journal.ppat.1009603)
Supplement: S3 Fig — In total, 77 ribosomal host proteins are displayed. SFV proteins are depicted in black circles and host proteins are displayed in smaller colour-coded circles according to the key. SFV-host PPI are depicted with solid grey lines. Grey boxes reflect host proteins that were identified as interactors to more than one of the SFV proteins. In these cases, the solid grey lines connect the grouped set of host proteins to the SFV proteins for which they were identified as interactors. Host-host PPI ascertained through STRING analysis are depicted with dotted pink lines. The host interactors were collected based on three independent biological replicates. SFV-ribosomal host protein interactors identified through a genome-wide fluorescence microscopy based siRNA screen as exhibiting potential proviral roles in SFV replication are additionally shown (turquoise borders, II ≤ 0.5). None were identified as having a potential antiviral role (Threshold ≥ 1.3). (PDF) [file ppat.1009603.s003.pdf]

Supporting information: Fig S3

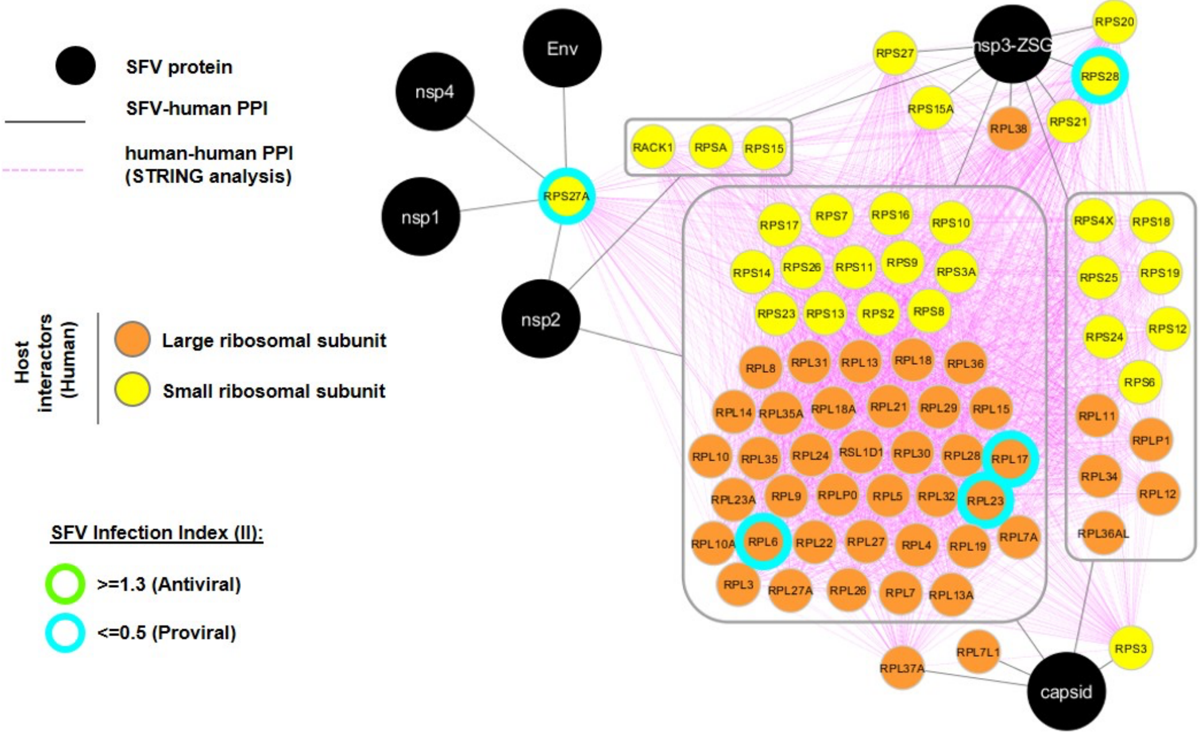

Figure S3. A network visualisation of the SFV-ribosomal host protein interactome. In total, 77 ribosomal host proteins are displayed. SFV proteins are depicted in black circles and host proteins are displayed in smaller colour-coded circles according to the key. SFV-host PPI are depicted with solid grey lines. Grey boxes reflect host proteins that were identified as interactors to more than one of the SFV proteins. In these cases, the solid grey lines connect the grouped set of host proteins to the SFV proteins for which they were identified as interactors. Host-host PPI ascertained through STRING analysis are depicted with dotted pink lines. The host interactors were collected based on three independent biological replicates. SFV-ribosomal host protein interactors identified through a genome-wide fluorescence microscopy based siRNA screen as exhibiting potential proviral roles in SFV replication are additionally shown (turquoise borders, II ≤ 0.5). None were identified as having a potential antiviral role (Threshold ≥ 1.3).
