# Supplementary material for: Characterisation of the Semliki Forest Virus-host cell interactome reveals the viral capsid protein as an inhibitor of nonsense-mediated mRNA decay
Source: PLoS Pathog. 2021 May 21;17(5):e1009603. doi: 10.1371/journal.ppat.1009603 (PMC8174725; doi:10.1371/journal.ppat.1009603)
Supplement: S4 Fig — SFV infection inhibits NMD at 3–4 hours post infection. RT-qPCR analysis showing mRNA levels relative to Actin-β of the viral RNA (first graph–‘ZSG’), NMD targets (black lines) and non-NMD targets (light grey lines) normalised to either the 0.5 hr sample (in the case of the viral RNA ‘ZSG’) or the Uninfected control, at different time points post SFV-ZSG infection. Cells were infected with an MOI of 10. Line graphs display the mean ± SD of four independent biological replicates (n = 4). Statistical significance was determined using unpaired, two-tailed t-tests, corrected for multiple comparisons using the Holm-Šídák method, with alpha = 0.05 (GraphPad Prism, v8.4.1). Asterisks indicate adjusted p values: p≤0.05*, p≤0.01**, p≤0.001***. (PDF) [file ppat.1009603.s004.pdf]

Supporting information: Fig S4

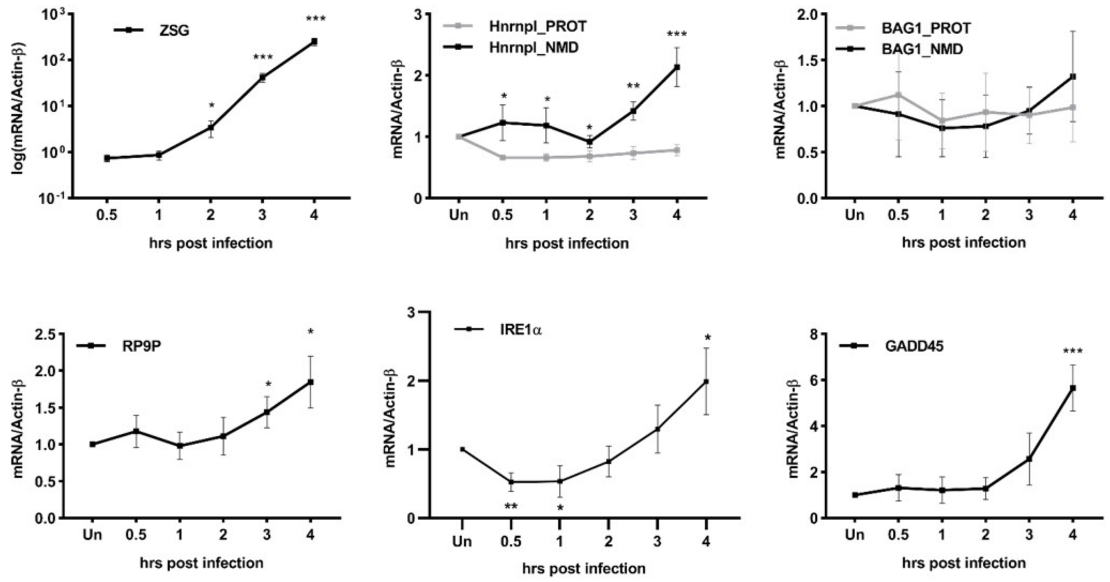

Figure S4 (Related to Figure 5). SFV infection inhibits NMD at 3-4 hours post infection. RT-qPCR analysis showing mRNA levels relative to Actin-β of the viral RNA (first graph – 'ZSG'), NMD targets (black lines) and non-NMD targets (light grey lines) normalised to either the 0.5 hr sample (in the case of the viral RNA 'ZSG') or the Uninfected control, at different time points post SFV-ZSG infection. Cells were infected with an MOI of 10. Line graphs display the mean ± SD of four independent biological replicates (n=4). Statistical significance was determined using unpaired, two-tailed t-tests, corrected for multiple comparisons using the Holm-Šídák method, with alpha = 0.05 (GraphPad Prism, v8.4.1). Asterisks indicate adjusted p values: p≤0.05*, p≤0.01**, p≤0.001***.
