# Supplementary material for: Characterisation of the Semliki Forest Virus-host cell interactome reveals the viral capsid protein as an inhibitor of nonsense-mediated mRNA decay
Source: PLoS Pathog. 2021 May 21;17(5):e1009603. doi: 10.1371/journal.ppat.1009603 (PMC8174725; doi:10.1371/journal.ppat.1009603)
Supplement: S5 Fig — SFV capsid expression did not alter polysome profiles or rRNA. a, HeLa cells expressing ‘empty’ (pcDNA5_FRT_TO_3xFlag) or ‘capsid’ (pcDNA5.3xFLAG-capsid) plasmids were treated with CHX [100 μg/mL], lysed and ultracentrifuged (274 000 x g, 2 hours, 4°C) through a 15–50% sucrose-CHX gradient to separate ribosomal subunits and monosomes from active/translating polysomes (Protocol adapted from [78]). The gradients were fractionated and the absorbance values (254 nm) throughout the gradient is plotted. b, Bioanalyzer analyses of RNA extracted from cytoplasmic (CYTO) and nuclear (NUCL) fractions of ‘empty’ or ‘capsid’ expressing cells. The peaks at ~42 and 49 [s] indicate the 18S and 28S rRNAs, respectively. RIN values indicate RNA integrity, 10 being the highest. c, RT-qPCR analysis showing raw CT values of 28S and 45S rRNAs from ‘empty’ or ‘capsid’ expressing cells. (PDF) [file ppat.1009603.s005.pdf]

Supporting information: Fig S5

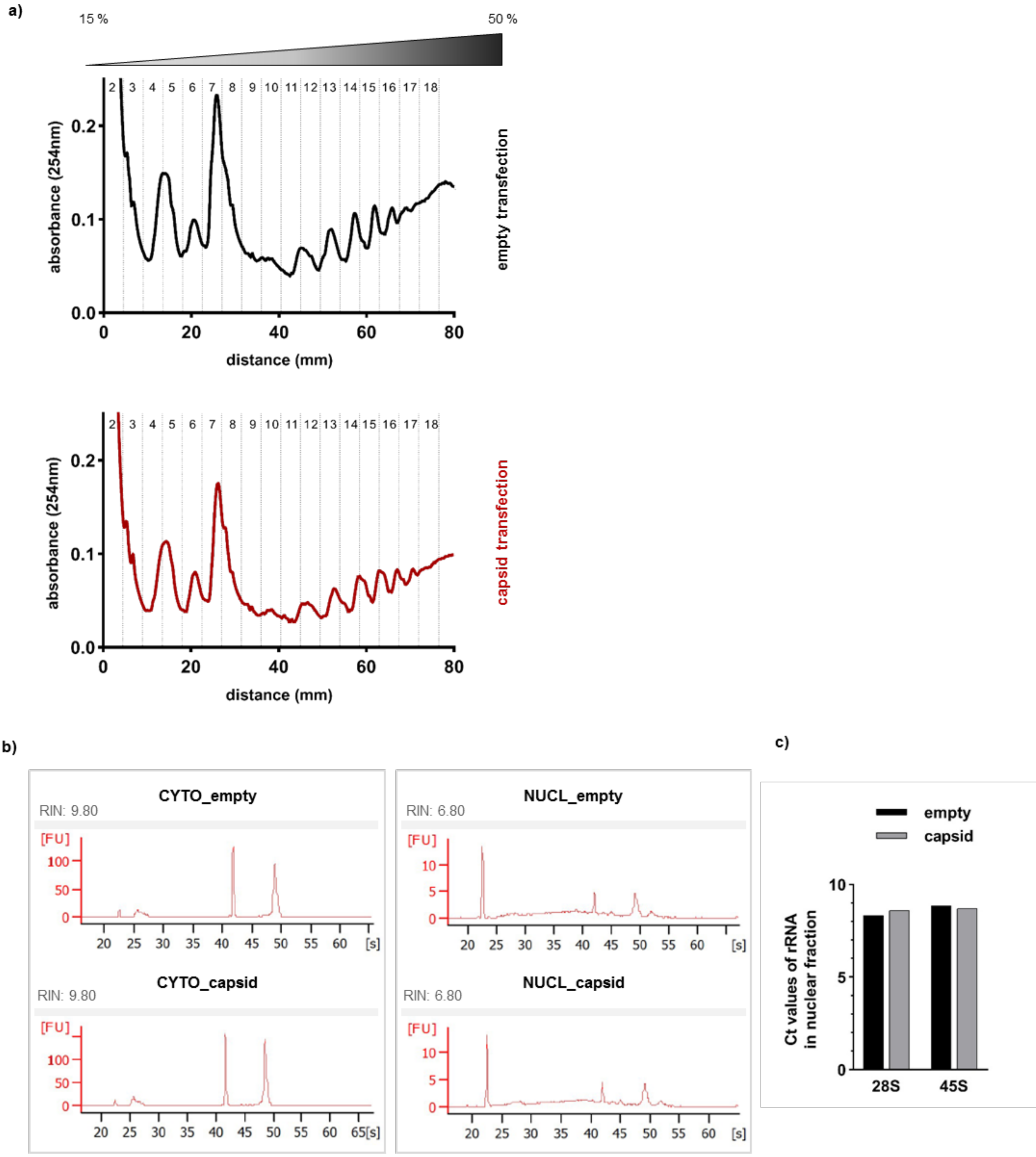

Figure S5 (Related to Figure 6). SFV capsid expression did not alter polysome profiles or rRNA. . a, HeLa cells expressing ‘empty’ (pcDNA5_FRT_TO_3xFlag) or ‘capsid’ (pcDNA5.3xFLAG-capsid) plasmids were treated with CHX [100 µg/mL], lysed and ultracentrifuged (274 000 x *g*, 2 hours, 4°C) through a 15-50 % sucrose-CHX gradient to separate ribosomal subunits and monosomes from active/translating polysomes (Protocol adapted from [78]). The gradients were fractionated and the absorbance values (254 nm) throughout the gradient is plotted. **b,** Bioanalyzer analyses of RNA extracted from cytoplasmic (CYTO) and nuclear (NUCL) fractions of ‘empty’ or ‘capsid’ expressing cells. The peaks at ~42 and 49 [s] indicate the 18S and 28S rRNAs, respectively. RIN values indicate RNA integrity, 10 being the highest. **c,** RT-qPCR analysis showing raw CT values of 28S and 45S rRNAs from ‘empty’ or ‘capsid’ expressing cells.
