# Supplementary material for: Characterisation of the Semliki Forest Virus-host cell interactome reveals the viral capsid protein as an inhibitor of nonsense-mediated mRNA decay
Source: PLoS Pathog. 2021 May 21;17(5):e1009603. doi: 10.1371/journal.ppat.1009603 (PMC8174725; doi:10.1371/journal.ppat.1009603)
Supplement: Attachment — Submitted filename: Respones to reviewers comments_MD_LC_OM_GiB.pdf [file ppat.1009603.s009.pdf]

Point to point response to reviewers' comments

Part I – Summary

Reviewer #1: The authors cataloged host proteins that bind to each of the expressed SFV proteins, identified host proteins showing pro- and anti-viral functions by using siRNA screening, showed SFV replication-induced inhibition of the NMD pathway late in infection and capsid-induced inhibition of the NMD pathway.

Although there is a possibility that all of the detected interactions may not occur in infected cells, their data will serve as a foundation for further understanding of interactions between host proteins and viral proteins in alphavirus-infected cells.

The biological significance of the capsid-induced inhibition of the NMD pathway on SFV replication was not experimentally explored. Does expression of capsid protect SFV RNA from NMD or rapid decay? Inclusion of data addressing this question would strengthen the significance of the present study.

Authors' response: The biological significance of capsid-induced inhibition of NMD is indeed not entirely clear. Given that the SFV genomic RNA (vRNA) is targeted by NMD upon entry into the cell (Balistreri *et al.*, 2014) and capsid is only expressed later during the infectious cycle from the sub-genomic (sg)RNA, we hypothesize that the capsid may protect newly synthesized full length vRNA or sgRNAs from the NMD pathway and so increase virus production.

Reviewer #2: The manuscript from Contu et al. describes the characterization of the Semliki Forest Virus (SFV)-host interactome and reveals a role for SFV capsid protein in the suppression of nonsense mediated decay (NMD). The authors utilize over-expression of individual SFV proteins followed by affinity purification to isolate viral-host protein complexes. Composition of these complexes was determined by quantitative mass spectrometry to identify interactors. Inclusion of RNase in sample preparation allowed the determination of those interaction that were dependent on RNA. Interaction networks were described on the basis of viral interacting partner (Fig 3) and on the basis of host cell gene ontology descriptions (Fig 4). The authors found numerous host proteins that interacted with multiple viral proteins, and enrichment of interacting partners in particular cellular functions, of note ribosome biogenesis, RNA binding and processing. Some of these had not been found in previous studies, but a listing of newly identified interactors did not appear in a figure. In parallel to the proteomic analysis the investigators performed a genome wide siRNA screen utilizing a SFV expressing a reporter GFP to assess both pro- and anti-viral effects. A proportion of interactors were found to have an impact on viral replication. Components of the NMD pathway were found to have an antiviral function which is consistent with previous studies from this group showing SFV to be susceptible to restriction by NMD and functionally inhibit NMD to facilitate efficient replication. Interaction with NMD was determined for each individual viral protein through the examination of known cellular NMD target transcripts. Expression of capsid protein increased the abundance of NMD target transcripts while not altering the abundance of control transcripts. Overall, the manuscript is clearly written, the mass spec analyses seem rigorous, as does the interactor analysis. The datasets will be of use to the broader field, and the finding that capsid inhibits NMD is significant.

Authors' response: We thank the reviewer for the succinct summary of our work and the overall positive assessment of the data analysis and the writing. The list of the identified host proteins interacting with the tested viral proteins is provided as Table 1. Rather than highlighting which of these are novel (which is the vast majority), we decided to mention instead in the paper those that have been previously described and cited the respective papers.

Reviewer #3: There is a growing understanding that viruses use diverse strategies to control the cellular translation and RNA decay machinery. In this manuscript, Contu and colleagues use proteomics to investigate host proteins that negatively or positively regulate Semliki Forest virus replication. Comparison of spectrometry of proteins associated with transiently expressed viral proteins to results of an RNAi screen identified many proteins involved in translation, ribosome biogenesis, and other aspects of RNA metabolism. Of these, the authors focus on UPF1 and the process of nonsense-mediated decay, showing that viral infection inhibits translation and NMD with similar kinetics. In addition to the likely indirect effect of translation inhibition on NMD, they also find that overexpression of capsid, but not several other SFV proteins, causes increased expression of several NMD target mRNAs, without an apparent effect on translation. The experiments are carefully performed, and the manuscript is well written. The apparently specific, translation-independent effect of capsid protein on NMD is of particular interest, although more insight into the mechanism of this inhibition would be welcome.

Authors' response: We thank the reviewer for the overall positive assessment of our work and agree that the capsid-induced NMD inhibition is the most interesting finding of this study, besides that the virus-host cell interactome provides a very useful resource for the research community.

Part II – Major Issues: Key Experiments Required for Acceptance

Reviewer #1: The authors stated that interactions of nsP3-Z and capsid with UPF1 is RNA-dependent. As experiments testing interactions of UPF1 with nsP3-Z and capsid shown in Fig. 6d were done without RNase treatment, this statement lacks solid experimental data. The authors should perform co-immunoprecipitation analysis shown in Fig. 6d by using cell extracts that are treated with RNase.

Authors' response: We assume that the reviewer refers to the data shown in Fig. 5d rather than 6d. These IPs were indeed done without RNase treatment to capture also the RNA-dependent interactions. However, our statement in the first sentence of the figure caption relates to the fact that in the reverse IPs (data shown in Fig. 2) pulling at nsp3-Z or capsid, UPF1 was only detected when IPswere not treated with RNase but not in the RNase-treated samples. That the interaction between capsid and UPF1 is RNA-dependent can also be seen in the UPF1-IP shown below in response to a point raised by reviewer 3 (see below, Fig. 1). However, we agree that the RNA-dependence has not been in the data shown in Fig. 5 and we therefore deleted "in an RNA-dependent manner" from the legend of Fig. 5 as well as "RNA-mediated" from the text (page 14, line 25).

Reviewer #2: no additional experiments, however some further data analysis:
1. The authors reference having confirmed interactions found in previous studies, and having found new interactors. It would be helpful to specify how many of the newly identified interactors have pro- or antiviral function. My main concern is that, in contrast to similar previous studies, the authors are expressing individual viral protein rather than examining the interactions in the context of an infection. While I believe the data generated to be of value, this approach does lend itself to the possibility of aberrant interactions that are not relevant in the context of an infection. Presentation of the number of these new, previously unrecognized, interactors with an impact on viral replication would counter this issue.

Authors' response: We agree with the reviewer that the expression of individual FLAG-tagged viral proteins has the limitation that in comparison to the interactions occurring during an infection, this method will miss interactions that depend on the presence of a complex formed by more than one viral protein and will produce some aberrant interactions due to the addition of the FLAG tag or the availability of interaction surfaces that during an infection would be covered by other factors. However, to determine the virus-host cell interactome during a real infection is technically not possible because of the lack of specific and for IP suitable antibodies against the viral proteins, and hence the approach taken by us represents a useful, valuable and technically amenable substitute that will identify a large fraction of the interactions that also occur during an infection. In addition, many viral proteins also exhibit individual functions that are separate from their functions within viral protein complexes. This strategy is therefore advantageous as it can help elucidate novel interactions that could be masked or confounded during the complex nature of an infection. The comparison of the identified host cell proteins that co-IPed with the respective transiently expressed FLAG-tagged viral proteins with those showing a functional (pro- or antiviral) effect in the siRNA screen is difficult for several reasons (see reply to next point) and calculating a number for the overlap of the hits of both assays is not meaningful, as both assays (especially the siRNA screen) are not saturating. Nevertheless, there are of course individual hits of the mass spec interactome mapping which exhibited an effect in virus replication in the siRNA screen. For example, novel interactions detected for the anti-FLAG capsid IP included a number of factors involved in rRNA processing /ribosome biogenesis that displayed anti-viral activity, including NOP58, NOP56 and FBL. Notably, NOP58 was **the** top anti-viral hit in the siRNA screen (Table 2). Another striking example is the newly identified interactor, SFPQ. As well as exhibiting pro-viral activity, SFPQ was identified as one of the most abundant interactors of both nsP2 and the most abundant interactor of capsid (Figure 2c).

2. The authors should address in some way why only a small handful of the interactors have an impact on virus replication. In looking at the siRNA spreadsheet it appears that there are over 3000 of the 38,000 knockdowns that meet the threshold for pro- or antiviral. Of the 251 interactors it appears that 30 (?) have an impact on viral replication. Is this level of enrichment of factors impacting replication in a set of interacting proteins what would be expected? The proportion of interactors impacting viral replication should be clearly stated. An explanation of why more identified interactors do not impact replication would be helpful. The low number of interactors influencing virus becomes very obvious in figure 4 where the interaction nodes shown have very few factors with pro- or antiviral effects, this should be addressed.

Authors' response: This is related to point 1.) raised above. We would argue that a quantitative statement regarding the overlap between the host cell interactors found to co-IP with the viral proteins and the host cell proteins exhibiting a pro- or anti-viral effect in the siRNA screen is not informative and potentially misleading, because this number is determined mainly by technical issues, such as for example on the exact criteria applied in the mass spec and the thresholds applied in the siRNA screen to call something a pro- or anti-viral effect. Thus, it would be a bit like a comparison between apple and oranges.

That overall fewer hits were identified in the siRNA screen than in the interactome mapping is to be expected and can be attributed to the following: (i) the siRNA screen produces many false negative results due to ineffective knockdown efficiencies and redundancies (two proteins with redundant functions would only show an effect if both were knocked down together), (ii) one would predict that only a fraction of the interactions between viral and cellular proteins affects the expression level of nsp3-ZsG, which was the readout in the siRNA screen; some interactions are expected to have other functional effects that are not resulting in an altered ZsG signal and yet others have no functional consequences at all.

Reviewer #3: 1. The major drawback of the study is a lack of insight into why the capsid protein interferes with turnover of NMD target mRNAs. The authors look for changes in polysome profiles and rRNA processing, but have they instead investigated whether capsid overexpression interferes with interactions between UPF1 and other proteins involved in NMD or NMD target RNAs?

Authors' response: We agree with the reviewer that it would have been desirable to gain some mechanistic insight into how capsid inhibits NMD. We have tested a number of hypotheses in our lab but unfortunately only obtained negative and hence ultimately inconclusive results. In addition to finding no differences in the polysome profiles and rRNA processing, we have also tested if capsid overexpression affected the association of UPF1 with mRNAs (both NMD sensitive and insensitive ones) by performing UPF1-RIP experiments but found no significant difference. In the course of revising this manuscript, we furthermore assessed if capsid overexpression affects the phosphorylation level of UPF1, which is a good proxy for NMD activation. To this end, we expressed in HeLa cells the capsid protein for 24 or 48 hours and immunoprecipitated from the cell lysate, which was either treated (+) or not with RNase A (–), UPF1 using an anti-UPF1 antibody (Figure 1). The IPed proteins were subjected to western blotting, probing with antibodies to detect phospho-UPF1 (P-UPF1, red channel), total UPF1 (green channel) and capsid (green channel). The first 4 lanes on the left side of the blot represent input (lysate before IP) and show increasing capsid levels over time. The co-IP of capsid with UPF1 is mainly RNA-dependent, as it is lost upon RNase A treatment at the 24h timepoint and strongly reduced at the 48h timepoint. Most importantly, the phosphorylation level of UPF1 is not affected by the presence of capsid protein, even when capsid is highly abundant in the cells 48h after transfection. Additionally, the UPF1 IPs showed no changes in the abundance of the well-known UPF1 interaction partner UPF2/RENT2 upon capsid expression (Figure 2; UPF2 is labeled as RENT2). However, since capsid is the 5th most abundant protein that co-precipitated with UPF1, and since it binds RNA, the presence of capsid on UPF1 containing mRNPs likely contributes to the NMD inhibitory phenotype. Nevertheless, despite all efforts,the mechanism by which capsid inhibits NMD remains unclear and will be addressed in the future.

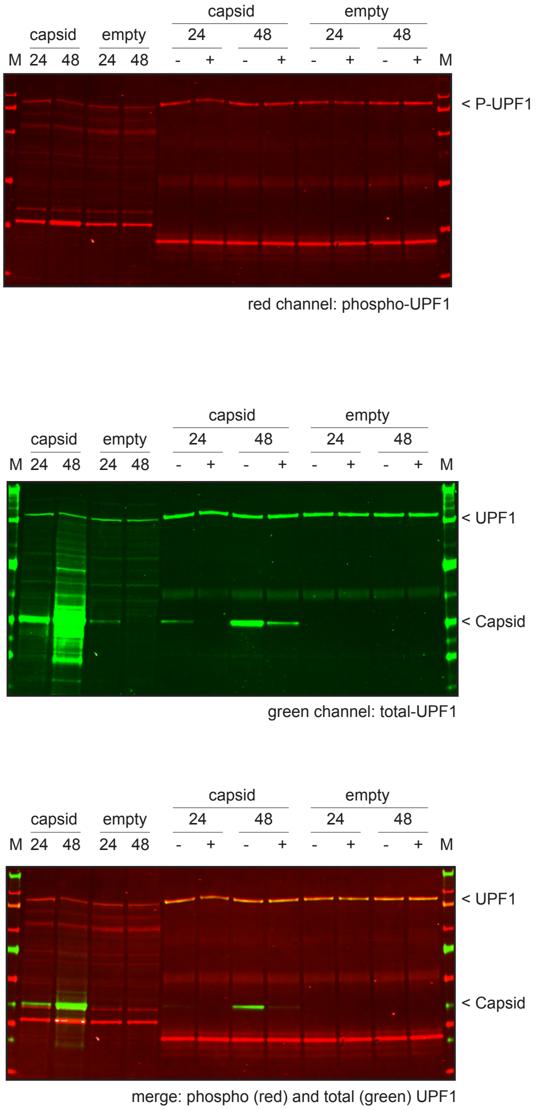

Figure 1: The extent of UPF1 phosphorylation remains unchanged upon capsid expression.

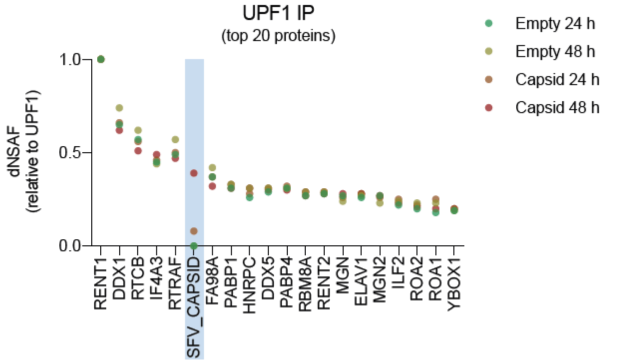

Figure 2: UPF2/RENT2 co-IP with UPF1 remains unchanged upon capsid expression.

2. Conversely, have the authors tried to use ISRIB or a PKR-deficient cell line to test whether there is evidence that viral infection inhibits NMD independent of translational repression? Further evidence for the physiological role of capsid-mediated NMD inhibition outside of the context of individual viral protein expression would be very helpful to establish the significance of the findings.

Authors' response: Prompted by the reviewer's suggestion, we used ISRIB in order to try to uncouple the NMD inhibition effect of capsid from the possible indirect NMD inhibition due to the translation inhibition occurring at later stages of the SFV infectious cycle. As shown in Figure 3, treatment of the cells with ISRIB (200 nM) efficiently prevents the translation inhibition that otherwise is seen 4 hours post infection. Regarding the effect on endogenous NMD sensitive mRNAs, the result is not entirely conclusive. It suggests that a part of the increased levels of endogenous NMD sensitive mRNAs observed at 4 hours post infection is caused by the translation shut-off (which is not unexpected) and another part (the smaller increase still detected in the ISRIB treated cells) presumably originates from the capsid-mediated NMD inhibition. Since we were still unable to unambiguously pull apart the source/s of NMD inhibition during infection, we decided not to include this new data in the manuscript.

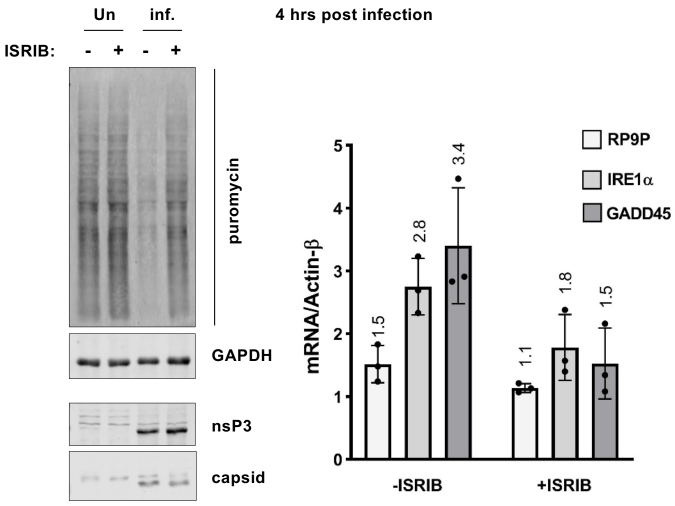

Figure 3: ISRIB prevents host translation shut-off 4 hours post infection, which leads to a partial reversal of the observed NMD inhibition at that time point. The remaining NMD inhibition can be attributed to capsid-mediated NMD inhibition.

3. Because of the central importance of establishing that capsid overexpression does not inhibit translation, quantification of the puromycylation assay in Figure 6d should be provided.

Authors' response: The puromycylation data has been quantified and the figure and figure legend has been modified accordingly (Figure 6d, lower panel).

Part III – Minor Issues: Editorial and Data Presentation Modifications

Reviewer #1: 1. Experiments shown in Fig. 1 included treatment of samples with RNase A. How did the authors know that the RNase treatment condition was appropriate? They should include data demonstrating that the experimental approach for RNase A treatment (page 23) were appropriate for RNA degradation.

Authors' response: The silver-stained gels of the eluates from the IPs of the different viral proteins show in all cases that most of the bands are lost when the IPs were treated with RNase A (Fig. 2a) and as expected, the interaction with known RNA binding proteins was lost (see. e.g. PABPC1 for nsp3-Z; hnRNPA1 for nsp2; SFPQ and Nono for capsid; Fig. 2c).

2. They claimed that expressed SFV proteins, including capsid and nsP3-Z, did not suppress translation nor induce eIF2a phosphorylation (Fig. 6), and concluded that capsid-induced NMD inhibition was not due to translational suppression. This conclusion was based on the assumption that capsid was expressed in most of the cells, yet this assumption was not experimentally tested. The current data do not exclude the possibility that only low levels of cells expressed capsid, which might have suppressed translation, leading to NMD inhibition. To exclude this possibility, they should show the percentages of cells expressing each of the viral proteins.

Authors' response: Transient transfections of HeLa cells with mammalian expression plasmids, such as the ones used for expressing the viral proteins, is a well-established standard procedure in our lab that results in expression of the respective transgenes in 40-70% of the cells (based on results obtained with fluorescent fusion proteins). There is no reason to assume that the percentage of cells expressing the viral proteins in this study would be lower. It should also be noted that during an SFV infection, we observed an increase of our endogenous NMD reporters of 1.5 to 6-fold (Fig. 5) and in the capsid overexpression these reporters increased 2 to 3-fold (Fig. 6), which we consider to be in a similar range.

3. Page 4. They used nsp3 carrying ZSG tag for protein expression, whereas no explanation about this tag was given at the beginning section of "Results". The authors should explain this tag and a rationale for the insertion of this tag within the nsp3 gene in first paragraph or early section of "Results" section.

Authors' response: The rationale for using ZsG-tagged nsP3 for the IPs was that the recombinant SFV used for the viral infections in our study and in many of the older studies also contains this ZsG tag in nsP3. Upon the reviewer's suggestion, we now better explained that the recombinant SFV used in previous studies contained a ZsG-tag inserted into nsP3 by changing in the last part of the Introduction (p. 4, lines 5-6) "nsP3-tagged recombinant virus" to "recombinant virus in which nsP3 was tagged with the fluorescent protein ZsGreen (ZsG). In the Results section, we then clarified the rationale for using nsP3-Z rather than nsP3 alone by adding on p. 5, lines 20-21: "In the case of the nsP3 bait (here fused with ZsG as in the recombinant virus, nsP3-Z)".

Reviewer #2: Some justification for choosing 1.3 and 0.5 as the II cutoffs is needed. Is this just arbitrary or is there a reason these values were chosen?

Authors' response: The two values correspond to an increase (1.3) and decrease (0.5) in infection levels. These thresholds were chosen because they correspond to the fraction of data that is >1.5 times the standard deviation of the whole screen (calculated from 10'000 scrambled controls distributed across the 96 well plates included in the screen).

While the authors focused on the interactors in the RNA containing samples, it is hard to know what to make of the changes in interactors in samples treated with RNase. The samples have no viral RNA present so RNA dependent interactions are not viral RNA dependent interactions, this should be clearly stated. Also, should any relevance be placed in the difference in interaction in the absence of RNA? This seems a very artificial situation.

Authors' response: The purpose of comparing IPs of RNase treated and untreated samples was simply to distinguish RNA-mediated from protein-mediated interactions. We think that this is especially informative in the case of RNA binding viral proteins (such as for example capsid), as it allows the distinction between co-precipitated host proteins that associate with the same RNA molecules (they are lost upon RNase treatment) from host proteins that might associate with the viral protein in a protein complex (these interactions are RNase insensitive).

Page 6, lines 6-9: Why does the fact that heat maps and silver-stained gels are consistent provide confidence in the approach to sample acquisition? These are just two different analyses of the same samples. Surely this provides confidence in the two techniques for analysis as they are consistent with one another.

Authors' response: We changed this sentence to "The quantified lists obtained to create the heat map were overall consistent with the patterns observed on the silver-stained gels", deleting the statement about confidence.

How do siRNA knockdowns impact cell viability? Is there some inherent within the analysis that accounts for siRNA induced cell death?

Authors' response: Toxicity was estimated based on the number of cells 72 h after in each siRNA treatment. Assuming that siRNA treatments that block cell division could result in a decrease in cell number up to 75% (if cells divide once per day for three days), this decrease value was chosen as the threshold of toxicity; siRNAs that decreased cell number more than 75% compared to scrambled controls were not considered.

Page 14, line 22 and Fig 5d: nsP2 was shown through MS to interact with UPF-1 but does not in 5d, conversely nsP1 was not seen to interact with UPF-1 by MS but does in 5d. Explanation?

Authors' response: The reviewer is correct in noting that in the eluate of the UPF1 IP, nsP1 was present but nsP2 was not detected, while UPF1 was detected in the eluate of the nsP2 IP but not in the nsP1 IP. It is frequently observed and due to technical limitations of the assay that IP-MS interactions are not always detected in the reciprocal IP-MS. In such cases, we did not further follow up the hit with functional experiments. Noteworthy and documenting the abovementioned variabilities inherent to IP-MS assays, peptides originating from UPF1 were in fact detected in the MS data of the nsP1 but based on our criteria applied in the SAINT analysis (abundance and coverage), it did not score as a "true interactor". Furthermore, nsP2 was detected in the RNase treated IP-MS (which we did not include in the manuscript) but not in the sample that was not treated with RNase.

Page 16, line 14-15: There's no reason not to show data, please add it.

Authors' response: Following the reviewer's suggestion, we have added this data as Suppl. Figure 5, panels b and c and adjusted the figure legend accordingly. We have also adapted the sentence describing this data on p. 16,

lines 12-15 (see below, response to reviewer #3, 2.). Methods related to this data have been included under the heading “Subcellular Fractionations and rRNA analysis” (page 32).

Page 25-26: For the MCODE method explanation Fig 5 is referenced and I believe this should be Fig 4.

Authors’ response: Thanks for spotting this mistake, we corrected it.

Reviewer #3: 1. Figure 4: The authors provide direct evidence of NMD compromise, but I think the GO analysis is somewhat misleading. The NMD go-term contains all of the ribosomal proteins, which means that any experiment in which ribosomes are enriched will lead to apparent enrichment for "NMD". This is compounded by the fact that for the purposes of GO analysis, ribosomal proteins are treated as independent entities, despite the fact that they are often obligate members of pre-ribosomes/ribosomes. From the supplemental table, it appears that only UPF1 among recognized NMD proteins (along with PABPC1, an inhibitor of NMD) contributes to this classification.

Authors’ response: The reviewer is correct, the GO term “NMD” contains indeed the ribosomal proteins, which certainly contributed to the high score of this term in our analysis. It can indeed be perceived as misleading that the Gene Ontology consortium included RPs in the “NMD” term and it might be wise to feedback to them to change this, but we would not feel comfortable to change the protein lists of individual GO terms for our own analysis, as this then would prevent a comparison with other GO term analyses of other publications. Despite this, the central NMD factor UPF1 was also identified in our IP-MS dataset (but no additional NMD factors) and for capsid, we showed in Fig. 6 that it indeed affects NMD. Thus overall, that the GO term “NMD” emerged from our interactome study was not misleading but by contrast led us into the right direction to discover that capsid inhibits NMD.

2.Page 16, last sentence of discussion: It's not clear what is meant by the sentence, "Though capsid could be trapped in the nucleus upon blocking of export…"

Authors’ response: This sentence was indeed unclear. We have modified it as follows: “However, we did not find any indications for capsid-induced defects in ribosome biogenesis or decreased ribosome abundance in capsid expressing cells: 18S rRNA, 28S rRNA and 45S precursor rRNA as well as polysome gradients were not altered (Suppl. Figure 5).”
